# Mineral composition in mussel *Mytilus galloprovincialis* and clam *Tapes decussatus* from Faro Lake of Messina: risk assessment for human health

**DOI:** 10.3389/ftox.2024.1494977

**Published:** 2024-12-13

**Authors:** Fabio Bruno, Vincenzo Nava, Sebastiano Zappalà, Giovanna Lucrezia Costa, Francesco Fazio, Vincenzo Parrino, Patrizia Licata

**Affiliations:** ^1^ Department of Veterinary Sciences, University of Messina, Messina, Italy; ^2^ Department of Biomedical and Dental Sciences and of Morphological and Functional Imagines (BIO-MORF), University of Messina, Messina, Italy; ^3^ Department of Chemical, Biological, Pharmaceutical and Environmental Sciences, University of Messina, Messina, Italy

**Keywords:** Bivalve, Faro Lake, minerals, *Mytilus galloprovincialis*, *Tapes decussatus*, risk assessment

## Abstract

Bivalve are exposed to a wide range of contaminants, some of which may be toxic to human health. The aim of this study was to detect essential and non-essential elements such as Na, Ca, Mg, Cr, Mn, Fe, Ni, Cu, Zn, As, Cd, Pb, Hg, Be and Co in water, sediments, and *Mytilus galloprovincialis* and *Tapes decussatus* from Faro Lake. It is a lake of marine origin located on the northern coast of Messina (Sicily), where shellfish farming has been practiced for many years. Metals were analysed by a single quadrupole inductively coupled plasma mass spectrometer (ICP-MS), except for Hg, which was quantified using a direct mercury analyser (DMA-80). The study evaluated the nutritional intake of elements through the ingestion of clams and mussels and the potential health risks to consumers. The lead levels found in *M. galloprovincialis* were below the LOQ, while in *T. decussatus* the concentrations were below the limit indicated by European Regulation 915/2023. Statistical analysis was carried out on *M. galloprovincialis* and *T. decussatus* samples using SPSS 27 and the data showed highly significant differences between the two species (*p* < 0.001). Cadmium (Cd) and mercury (Hg) concentrations were also below the legal limit in all samples analyzed. This study has shown that clams and mussels are a source of sodium (Na) with a Recommended Dietary Allowance (RDA) of 36% in *M. galloprovincialis* and 77% in *T. decussatus*. The percentages obtained for calcium (Ca) and magnesium (Mg) were 17%–19% and 18%–8%, respectively. The RDA of chromium (Cr) was of 191% for *M. galloprovincialis* and of 405% for *T. decussatus.* The Fe percentages were 92% and 169% for *M. galloprovincialis* and *T. decussatus*, respectively. The concentrations of the other metals observed in the two bivalve species of Lake Faro were generally lower than the Tolerable Weekly Intake (TWI) values estimated as a risk to human health.

## 1 Introduction

Bivalve molluscs represent a valuable source of nutrients for human health, including proteins, omega-3 fatty acids, vitamins and metal elements, such as selenium and calcium (Prato et al., 2019; Durazzo et al., 2022). However, different types of contaminants to which marine organisms are exposed could cause a risk to consumers (Álvarez-Muñoz et al., 2016). Essential elements play a vital role in the functioning of living systems, as they participate as coenzymes in enzymatic reactions, regulate membrane transport, and ensure nervous and muscular excitability, among other functions (Weyh et al., 2022). Indeed, fluctuations in the concentration of essential elements can lead to changes in normal physiological processes (Baudry et al., 2020). In the field of human nutrition, bivalves represent an excellent source of essential elements, and their determination is therefore useful for assessing the presence of metal contaminants, which indicate the toxicity of molluscs and environmental pollution (Kapranov et al., 2021; Vereycken and Aldridge, 2023). The trace and macro elements play an important biological activity in animals and in humans; sodium, together with the potassium, is involved in the synaptic communication processes and the maintenance of the cellular electrochemical gradient (Singh et al., 2011; Delaney and Finfer, 2014; Strazzullo and Leclercq, 2014). Calcium is useful for bone mineralisation, cellular communication, the neuromuscular junction function, and the cardiovascular system (Vannucci et al., 2018). Iron is involved in the O_2_ transport in haemoglobin and in electron transport in oxidative phosphorylation processes (Paul et al., 2017). Magnesium and zinc play key roles in the biosynthesis of fatty acids and proteins, in folding and genome processes, and in the regulation of immune cells (Thompson, 2022; Weyh et al., 2022). Copper, as an electron transporter, is involved in oxidative phosphorylation and connective tissue formation, while cobalt is essential for the relocation of methyl groups in DNA, as a component of vitamin B12, and in other roles (Ruiz et al., 2021; Genchi et al., 2023). Manganese has an antioxidant effect, and is a cofactor in cellular energy metabolism, in the growth of connective and skeletal tissues, while molybdenum is involved in the metabolism of uric acid (Sardesai, 1993; Avila et al., 2013). Essential and non-essential elements are found in coastal marine environments as a result of anthropogenic activities that alter the habitat of aquatic organisms (Kolarova and Napiórkowski, 2021). The toxicity of these elements depends on their varying degrees of persistence, bioaccumulation and biodegradation (Khawar et al., 2024). Contamination of the marine environment is primarily attributable to toxic elements that are easily absorbed and bioaccumulated due to their ability to interact with the carbon chains of organic compounds (Hama Aziz et al., 2023). The significant increase in consumption of bivalve molluscs in recent years has led to an increase in request and consequently in their production, with an increase of up to 18 megaton (Mt) of molluscs, mostly bivalves, with an income of 30 USD billion (FAO, 2022; FAO, 2024). However, their production is essentially based on the presence of natural quantities of marine phytoplankton and debris, transported by currents and tides (Wijsman et al., 2019). The economic importance of shellfish for people living close to coastal ecosystems is fundamental, but the economic income is limited by the resources invested in the production process and by the pollution of coastal systems (Figueira and Freitas, 2013). The market value of bivalves is estimated to be approximately 23 billion dollars per year (2010–2015) (Wijsman et al., 2019). However, the total value is likely to be considerably high, given the secondary services associated with their marketing, such as packaging, transport and prepared products (Economics, 2010). The *Mytilus galloprovincialis* mussel and *Tapes decussatus* clam are filter-feeding organisms, which can be used for biomonitor the aquatic habitat. The mussel *M. galloprovincialis* is a sessile bivalve with a wide distribution in the Mediterranean and Black Sea, primarily in temperate environments along rocky coastlines or on other structures that included stilts and submerged jetties (Peycheva et al., 2023a). Bivalve exhibit a high degree of filtration (2–4 L/h) and they showed a high degree of adaptability to variations in temperature and salty water (Vera et al., 2011). The living habitat of *T. decussatus* is represented by the Mediterranean Sea, although it is distributed in the British Isles and along the Atlantic coasts from Norway to Senegal (Lucrezia et al., 2011). These clams are benthic organisms with lower filtration rates (1 L/h) than mussels (Vera et al., 2011). They live semi-buried in the sand or muddy seabed of tidal flats and shallow coastal areas, including coastal lakes (Chícharo and Chícharo, 2001). Both species hold commercial relevance, due to their consumption on a large scale not only by the local population, but also through the international export in other countries (Dellali et al., 2021). Trace and macro elements, found in the marine environment as natural constituents, can be classified into essential metals such as copper, zinc, iron and manganese, probably essential metals like nickel and cobalt and toxic metals as arsenic, cadmium, lead and mercury (Muñoz-Olivas and Cámara, 2001). At low levels, essential elements are useful for enzyme activities and many biological processes, but are toxic when they are taken in excess, for kidney, liver, nervous system and lungs (Araújo and Cedeño-Macias, 2016; Dadar et al., 2016). This can occur through the ingestion of foodstuffs capable of biomagnification along the trophic chain, which involves first, the smallest organisms (such as filter-feeding molluscs) and then the larger ones, allowing the contaminants to become a health risk for consumers (Baeyens et al., 2005; Türkmen et al., 2008). Bivalve molluscs together with other organisms can constitute an efficient means of accumulating toxic contaminants released into the surrounding environment such as roads, cultivated fields, wastewater management facilities, workshops and shipyards, as well as fish farms (Mesquita et al., 2023). The exposure of organisms mainly involves multiple indirect contaminations related to interactions between environmental matrices, which cause the accumulation of toxic substances primarily in organisms at the base of the trophic chain and especially in marine habitats (Dellali et al., 2022). The occurrence of bioaccumulation and multiple exposure phenomena, along the trophic chain is a matter of interest to the European Community, which is committed to protect the public health by ensuring the food safety for consumers, with specific attention to children, elderly and ills. The European Union, with the enforcement of EC Regulation 915/2023, has set specific maximum limits on the concentration of several animal and plant food contaminants, including some metals (EC, 2023a).

The aim of this study was to determine Na, Ca, Mg, Cr, Mn, Fe, Ni, Cu, Zn, As, Cd, Pb, Hg, Be and Co concentrations in water, sediments and samples of *M. galloprovincialis* and *T. decussatus* from Lake Faro of Messina, Sicily. It is a natural water basin used for shellfish farming. In addition, in this research the nutritional intake through the ingestion of *M. galloprovincialis* and *T. decussatus* was evaluated, as the health risk for consumers. Furthermore, the essential element concentrations (Na, Ca, Mg, Cr, Mn, Fe, Ni, Cu and Zn) were compared with the Recommended Dietary Allowance (RDA) and the Adequate Intake (AI) values. The exposure to non-essential elements (As, Cd, Pb and Hg) was assessed by comparing the toxicity of the elements with the Tolerable Weekly Intake (TWI), Tolerable Daily Intake (TDI) or the lower confidence limit of the benchmark dose (BMDL01).

## 2 Materials and methods

### 2.1 Samples collection

During September 2023, 160 specimens of *Mytilus galloprovincialis* (80) and *T. decussatus* (80) were collected from various Lake Faro (ME) sampling sites, forming a single sample site. It is a lake of marine origin, located on the northern coast of Messina (Sicily). Its depth, about 30 m, makes it the deepest of both coastal and internal lakes in Italy. It communicates with the Tyrrhenian Sea and the Strait of Messina. The physiochemical parameters of the water were assessed using a pH meter. Samples of surface water and sediment were collected, placed in plastic bottles and refrigerated at 4°C in the laboratory. The WTW Multi 340i/SET multi-parameter sensor was utilized to measure temperature, pH, dissolved oxygen, and conductivity values in field and laboratory settings. Ammonia, nitrites, phosphate, and orthophosphate were also measured (data not showed). Samples taken straight from the lake were cooled at 4°C for 24 h prior to analysis.

### 2.2 Mineralization

Mineralization of soft tissues was carried out according to Bruno, et al. (2024) and 0.5 g of mussel sample was weighed in PTFE vessels. Each sample was digested in triplicate using a closed-vessel microwave digestion system Ethos 1 (Milestone, Bergamo, Italy). To each sample, 1 mL of internal Re standard at a concentration of 0.5 mg/L, 8 mL of HNO_3_ (65% volume/volume), and 2 mL of H_2_O_2_ (30% volume/volume) were added (Bruno et al., 2024). Table 1 shows the instrumental parameters and settings. Each sample was made up to volume using 25 mL of ultrapure water as a blank after digestion and cooling. All samples were filtered with 0.45 µm PTFE filters prior to ICP-MS analysis. For digestion and subsequent dilution of tissues samples, the following reagents were purchased from Fluka (Milan, Italy): hydrogen peroxide (H_2_O_2_, 30% v/v), nitric acid (HNO_3_, 65% v/v) and ultrapure water (Bruno et al., 2024).

**TABLE 1:** Microwave oven operative settings ingestion.

Step	Time	Temperature	Microwave power
1	18 min	0°C–175°C	1100 W
2	18 min	175°C	1100 W
3	22 min	Cooling	

### 2.3 ICP-MS and minerals detection

The commercial standard solution of Re (internal standard) and the standards of the elements utilized for the calibration curves were obtained from Supelco (Bellefonte, PA, United States). Thermo Scientific ICP-MS iCAP-Q was used to determine Na, Ca, Mg, Cr, Mn, Fe, Ni, Be and Co, and all the samples were analysed three times under the same conditions (Bruno et al., 2024). The following operating conditions were used to analyse all samples: RF power, 1,550 W; plasma gas flow rate (Ar), 15 L/min; auxiliary gas flow rate (Ar), 0.9 L/min; carrier gas flow rate (Ar), 1.2 L/min; collision gas flow rate (He) flow rate, 4.8 mL/min; spray chamber temperature, 2.8°C; sample depth and sample introduction flow rate, 5 mm and 0.94 mL/min. Integration times were 0.6 s/point for As and 0.2 s/point for the other elements. Data acquisition was performed using Thermo Scientific QtegraTM Intelligent Scientific Data System software, Waltham, United States. For quantitative analysis, a seven-point calibration curve with internal standard normalization was also constructed.

### 2.4 Statistical analysis

Statistical analysis was carried out using SPSS 27.1 (IBM Company, Novegro-Tregarezzo, Italy). Shapiro-Wilk normality was performed. Results were reported as mean ± standard deviation (SD). Differences between species were assessed using *t*-test for indipendent data. Data was corrected automatically by SPSS with the base 10 logarithm. Statistical significance was set at *p* < 0.05.

## 3 Results and discussion

### 3.1 Method validation

The ICP-MS and DMA-80 procedures were carried out by Eurachem requirements (Bertil et al., 2014). The methods were validated in terms of linearity (*R* ^2^), sensitivity (LODs and LOQs), and accuracy (% of recovery), as shown in Table 2. Six standard solutions were injected eight times each to check linearity. Calibration curves were generated for each analyte. All concentration ranges exhibited good linearity, with *R* ^2^ values consistently equal to or higher than 0.999. Experimental calculations yielded the following results for the limits of detection (LODs) and limits of quantification (LOQs): 3.4 s/S and 9.90 s/S, respectively (s is the standard deviation of the response of ten blanks and S is the slope of the calibration curve). The LOD and LOQ ranges were from 0.001 to 1.314 mg/Kg and from 0.003 to 4.336 mg/Kg respectively. Accuracy was assessed using the certified reference material, ERM-CE278k-mussel tissue. This parameter was evaluated by comparing six certified matrix determinations and the result was a percentage of recovery between the true value reported in the certified reference materials and the value found experimentally. The matrix was spiked with a known quantity of analyte and then examined if the element was not verified in the reference material. Linearity, sensitivity, accuracy, and precision were suitable for this analysis.

**TABLE 2:** Analytical parameters of the method.

Element	R2	LOD (mg/Kg)	LOQ (mg/Kg)	ERM-CE278k-mussel tissue		
				Experimental value (mg/Kg)	Certified value (mg/Kg)	Recovery (%)
Ca	0.999	0.016	0.053	1817.8	1830	99.33
Be	0.999	0.001	0.003	1.934	2.0^a^	96.70
Ni	0.999	0.004	0.013	0.688	0.69	99.71
Cr	0.999	0.001	0.003	0.71	0.73	97.26
Fe	0.999	0.011	0.036	158.3	161	98.32
Mg	0.999	0.028	0.092	1,461	1,510	96.76
Mn	0.999	0.011	0.036	4.82	4.88	98.77
Na	0.999	1.314	4.336	13,157	13,900	94.66
Co	0.999	1.140	3.762	0.20	0.21	95.24
Cu	0.999	0.013	0.043	5.95	5.98	99.50
As	0.999	0.001	0.003	6.5	6.7	97.02
Cd	0.999	0.001	0.003	0.339	0.336	100.89
Pb	0.999	0.001	0.003	2.21	2.18	101.38
Zn	0.999	0.033	0.109	68.95	71.00	97.11
Hg	0.999	0.001	0.003	0.068	0.071	95.78

^a^ Analyte not present in the certified matrix.

### 3.2 Essential and non-essential elements

The possible risks to human health from the consumption of these species may be due to several factors that influence the amounts of elements in mussels and clams, including diet, season and the possible environmental pollution (Mititelu et al., 2022). The elements found in *M. galloprovincialis* and *T. decussatus* samples are reported in Table 3.

**TABLE 3:** Elements in mussel *M. galloprovincialis*, in clam *T. decussatus*, in water and sediment samples from Faro Lake.

Element	*M. galloprovincialis* (mg/Kg)	*T. decussatus* (mg/Kg)	Water (mg/L)	Sediment (mg/Kg)
Na	3,564 ± 303	7,715 ± 59^a^	2.6 ± 0.1	9,532 ± 441
Ca	903 ± 0.4	1,005 ± 1^a^	1,022 ± 59	8,109 ± 37
Mg	443 ± 1.3^a^	203 ± 7	10.2 ± 0.7	9,612 ± 78
Cr	0.51 ± 0.14	1.1 ± 0.2^a^	<LOQ	36.39 ± 0.34
Mn	3.15 ± 0.07	7.1 ± 0.5^a^	0.11 ± 0.05	605 ± 0.54
Fe	86.2 ± 0.3	157.9 ± 0.5^a^	2.57 ± 0.08	10,827 ± 782
Ni	0.65 ± 0.08	1.77 ± 0.09^a^	0.22 ± 0.07	76.17 ± 0.08
Cu	3.1 ± 0.2^a^	1.30 ± 0.12	<LOQ	18.30 ± 0.3
Zn	16.5 ± 0.3^a^	4.22 ± 0.18	0.22 ± 0.02	85.20 ± 0.5
As	<LOQ	0.08 ± 0.01^a^	<LOQ	2.90 ± 0.32
Cd	0.05 ± 0.01	0.08 ± 0.02^a^	0.18 ± 0.04	0.39 ± 0.05
Pb	<LOQ	0.02 ± 0.01^a^	<LOQ	7.10 ± 0.09
Hg	0.01 ± 0.00	0.01 ± 0.00	<LOQ	0.03 ± 0.00
Be	<LOQ	<LOQ	<LOQ	1.27 ± 0.14
Co	0.11 ± 0.03	0.90 ± 0.01^a^	0.31 ± 0.06	5.77 ± 0.35

^a^ Differences between *M. galloprovincialis* and *T. decussatus* (*p* < 0.001). Data are expressed as mean ± standard deviation.

#### 3.2.1 Trace elements in Bivalve species

The mean concentrations (mg/Kg) and standard deviations of the elements of the two bivalve species from Lake Faro are shown in Table 3. Concentrations of Mn, Cr and Ni were higher in *T. decussatus* than in *M. galloprovincialis*, while Fe was higher in *M. galloprovincialis* than in *T. decussatus*.

Nickel is normally occurred at very low levels in the environment, but bivalves have a high filtration capacity, which lead to the accumulation of this and other different pollutants in the molluscs. This particular feature of filter-feeding organisms allows them to be used as “bioindicators” of the aquatic environment. High levels of Ni can cause skin allergies, chronic bronchitis, reduced lung function, and cancer of the lung and nasal sinuses (Hédouin et al., 2007). Nickel concentrations found in *M. galloprovincialis* (0.65 ± 0.08 mg/Kg) were lower than in *T. decussatus* (1.77 ± 0.09 mg/Kg). As reported in the literature, nickel concentrations ranged from 0.13 to 0.43 mg/Kg ww in *M. galloprovincialis* from the Italian coast and the western Adriatic Sea, but in a recent study conducted in the Black Sea (Bulgaria) by Peycheva et al. (2023b), nickel concentrations were up to 0.04–0.21 mg/Kg ww (Desideri et al., 2010). Chromium, manganese, and iron are essential elements for humans and most of them occur naturally in many food sources. The concentration of Cr was almost twice as high in clams than in mussels, with a Cr content of 0.51 ± 0.14 mg/Kg in *M. galloprovincialis* and 1.08 ± 0.17 mg/Kg in *T. decussatus*. Moreover, our results showed similar concentrations to those reported in the literature (Bilgin and Uluturhan-Suzer, 2017).

Manganese is well recognized as a necessary element for both plants and animals, so an inadequate amount of Mn can cause major damage to the skeletal and reproductive systems (Sivaperumal et al., 2007). The Mn concentrations found in *M. galloprovincialis* were of 3.15 ± 0.07 mg/Kg ww and in *T. decussatus* of 7.08 ± 0.50 mg/Kg ww. These values are in line with those reported in the literature for wild species of *M. galloprovincialis* from the Sea of Marmara (Bosporus), with levels ranging from 0.865 to 11.306 mg/Kg ww and from 1.33 to 3.85 mg/Kg ww for *M. galloprovincialis* in three locations of Boka Kotorska in 2015 (Özden et al., 2010; Perošević et al., 2018).

The iron levels detected in bivalve species were of 86.22 ± 0.27 mg/Kg for *M. galloprovincialis* and 157.91 ± 0.50 mg/Kg for *T. decussatus*. According to European legislation, there is no maximum level for Fe in bivalve (EC, 2023b), but the US Food and Drug Administration has set 80 mg/Kg ww as a limit (FDA, 2012) and a safe daily dose of 40 mg of Fe has been established for the adults, including pregnant or lactating women (EFSA et al., 2022). The iron values found in our bivalve samples may be due to fertilizers and pesticides used in the surrounding agricultural areas, but also to contamination of raw materials in the marine environment, such as bins and metal cases, poor handling practices in waste disposal, or boat maintenance procedures (Mehrandish et al., 2019; Byrnes and Dunn, 2020). Beryllium concentrations were below the LOQ. Co concentrations were found to be 0.11 ± 0.03 mg/Kg for *M. galloprovincialis* and 0.9 ± 0.01 mg/Kg for *T. decussatus*. These Co values are in accordance with those reported in the literature, with values ranging from 0.038 to 0.084 mg/Kg for *M. galloprovincialis* samples in the same research area (Meloni et al., 2022).

The Zn content was similar between the two species analysed with values of 16.52 ± 0.30 and 14.22 ± 0.18 mg/Kg in *M. galloprovincialis* and *T. decussatus*, respectively. Our results are higher than those reported by Ozden, et al. (2010) in *M. galloprovincialis*.

The amount of Zn undoubtedly comes from inappropriate incineration of municipal solid waste, and most of the Zn in the soil remains bound to solid particles. Zn dispersed in the air is absorbed by the soil and transported to large reservoirs where it settles to the bottom (Wei et al., 2023). Zn is then taken up by bivalve organisms.

The analysis of Cu in *M. galloprovincialis* reported a higher concentration than in *T. decussatus* with values of 3.11 ± 0.15 mg/Kg and 1.30 ± 0.12 mg/Kg respectively, due to the higher volumes of water filtered by the mussels. The copper levels are in line with those reported by Di Bella et al. (2013) in samples of *Cerastoderma edule glaucum* and *Venerupis aurea laeta* from Lake Ganzirri (Messina, Italy).

The *M. galloprovincialis* showed a concentration of As below the limit of quantification, whereas the *T. decussatus* reported a level of 0.08 ± 0.01 mg/Kg. The arsenic contents in the samples were in agreement with those found by Licata et al. (2004) for *M. galloprovincialis* from Faro Lake. Furthermore, the data obtained showed a higher concentration of As in the sediment than in the water, because there is a possible uptake in the benthic environment (Fendorf et al., 2008; Litzow, 2008). The bioavailability of As depends on the pH of the marine environment, as demonstrated by Fendorf et al. (2008) and Velez et al. (2016). However, the As concentrations found in mussel and clam samples were lower than those reported in the literature (Meloni et al., 2022).

Cadmium concentrations in *M. galloprovincialis* and *T. decussatus* were slightly lower (0.05 ± 0.01 and 0.08 ± 0.02 mg/Kg, respectively) than those found by Di Bella et al. (2010) in two different species of clams (*V. aurea laeta* and *C. edule/glaucum*) collected from Ganzirri Lake (Messina), with a range between 0.011 ± 0.001 and 0.044 ± 0.008 mg/Kg, respectively.

The lead levels found in *M. galloprovincialis* were below the LOQ, while in *T. decussatus* the concentrations were 0.02 ± 0.01 mg/Kg, slightly higher than in the mussel samples. This may be related to the living conditions of the bivalve *T. decussatus*, which lives in contact with the benthic sediments, compared to *M. galloprovincialis*, which is attached to rocks and reefs. Furthermore, Pb concentrations in the sediment were higher (7.10 ± 0.09 mg/Kg) than those found in the water, where the levels were below the limit of quantification value. As reported by Culotta et al. (2008), Pb content in *T. decussatus* samples from Ganzirri Lake in Messina (Italy) was higher than those described in our study (Culotta et al., 2008).

The mercury concentrations were like those showed in the literature by Castello et al. (2019) in *M. galloprovincialis* samples from different Sicily coasts. The results reported that the Hg concentrations in *M. galloprovincialis* and *T. decussatus* were equivalent to each other and, as reported in EC Regulation 915/2023, below the MRLs (EC, 2023b).

#### 3.2.2 Macro elements

Table 3 shows the concentrations of the elements analysed. Sodium and calcium were the most abundant elements in all samples. Sodium is usually one of the most abundant macro-elements in seafood, such as bivalves from salt or fresh water, as reported in the literature (Moniruzzaman et al., 2021). *M. galloprovincialis* showed lower Na levels (3,564.263 ± 302.96 mg/Kg ww) than to *T. decussatus* (7,715.37 ± 58.82 mg/Kg ww). Calcium concentrations were 903.27 ± 0.44 mg/Kg in *M. galloprovincialis* and 1,005.28 ± 1.02 mg/Kg in *T. decussatus*. A different trend was observed for Mg concentrations. In fact, the highest levels of this element were found in *M. galloprovincialis* (443.28 ± 1.31 mg/Kg ww), whereas the lowest in *T. decussatus* (203.17 ± 7.42 mg/Kg). However, the concentration range for these elements in the studied species was within the values reported by several authors (Özden et al., 2010; Nekhoroshkov et al., 2021).

#### 3.2.3 Statistics data

The levels of Fe, Na, Ca, As, Cd, Co, Cr, Mn, Ni, Pb were higher in *T. decussatus* than in *M. galloprovincialis.* These concentrations showed significant differences between the two analysed species (*p* < 0.001). Mg, Cu and Zn concentrations were higher in *M. galloprovincialis* than in *T. decussatus* (*p* < 0.001). No significant difference was found between the Hg concentrations in *T. decussatus* and *M. galloprovincialis* (*p* > 0.05). No concentrations of As and Pb were found in *M. galloprovincialis* (<LOQ), while Be levels were below LOQ in both species (Table 3).

#### 3.2.4 Human health risk assessment of mussels/clams consumption

For the European Food Safety Authority and the European Commission, the recommended daily allowances (RDAs) for Ca, Cr, Cu, Fe, Na, Mg, Mn and Zn are 800, 0.040, 1, 14, 1,500, 375, 2 and 10 mg/day, respectively (EFSA, 2005; ECC, 2008). The TDI and BMDL01 reference values are used for toxic and potentially toxic elements: As at 0.3 μg/Kg b.w./day (EFSA, 2009), Hg at 4 μg/Kg b.w./day (EFSA, 2012), Pb at 0.5 μg/Kg b.w./day (EFSA, 2010) and TWI reference values for Cd at 2.5 μg/Kg b.w./week (Arcella et al., 2012). An opinion on the public health risk of Ni in food and drinking water was adopted by the EFSA’s Scientific Panel on Contaminants in the Food Chain (CONTAM), which established a tolerable daily intake (TDI) of 2.8 μg/Kg Ni/Kg b.w per day (0.0028 mg/Kg Ni/Kg b.w or 0.0196 mg/Kg Ni/Kg b.w tolerable weekly intake) (EFSA et al., 2020). Table 4 shows the assessment of the mineral contribution derived from the consumption of mussels and clams gathered from various sampling sites in Lake Faro (Messina, Italy). To calculate the daily exposure (mg/d), the average concentration in the sample (mg/Kg) was multiplied by the amount consumed (g).

**TABLE 4:** Elements uptake (%) for *M. galloprovincialis* and *T. decussatus*.

	Elements uptake (%)												
	Na	Ca	Mg	Cr	Mn	Fe	Ni	Cu	Zn	As	Cd	Pb	Hg
*M. galloprovincialis*	36	17	18	191	24	92	6	42	23	—	24	—	1
*T. decussatus*	77	19	8	405	53	169	17	17	20	57	48	13	1

The estimation of the metal contribution from the consumption of 150 g/day of molluscs was based on the minimum and maximum values of the analysed samples (Table 4) (INRAN-CRANUT, 2014).

The highest uptake was mostly obtained for *T. decussatus*. The uptake of toxic elements was very low in all samples. Arsenic (As) and lead (Pb) uptake was only calculated for *T. decussatus* samples with a percentage of 7% and 3% respectively. This was not possible for *M. galloprovincialis* as the concentrations of these elements were below the limit of quantification. Cadmium (Cd) uptake ranged from 3% for *M. galloprovincialis* to 7% for *T. decussatus*, while for Hg the mussels and clams showed the same uptake percentages (1%). This study showed that clams and mussels were also a good source of sodium (Na), with an RDA ranging from 36% in *M. galloprovincialis* to 77% in *T. decussatus*. For calcium (Ca) and magnesium (Mg), the percentages obtained were of 17%–19% and 8%–18%, respectively (Table 4). Among the essential trace elements, chromium (Cr) and iron (Fe) uptake was the highest with percentages of 191%–405% and 92%–169% for *M. galloprovincialis* and *T. decussatus*, respectively.

The content of Cr was 191% for *M. galloprovincialis* and 405% for *T. decussatus*. These results require special attention due to the known effects of Cr (VI), classified by the International Agency for Research on Cancer (IARC) as a Group 1 carcinogen, and not of chromium (III), which is not a carcinogen but an essential nutrient whose deficiency can have negative effects on human health (DesMarais and Costa, 2019). However, since the ICP-MS analysis carried out determined the total Cr content, we cannot determine the exact form of this element. For this reason, it is crucial to continuously monitor the Cr concentration in clam and mussel samples analysed for the protection of consumer health. Gastrointestinal risks related to the presence of Cr (VI) are another concern related to the chromium intake through the mussel consumption. The metal can irritate the digestive system, causing symptoms such as nausea, vomiting and gastrointestinal disorders. Furthermore, the presence of chromium in water can have a negative impact on soil quality and on the food chain, thus contributing to further risks to human health. Cr (III) can also have adverse health effects: although it is considered less toxic than Cr (VI), long-term accumulation of trivalent chromium can contribute to health problems such as liver and kidney damage. The presence of Cr in the waters of Faro Lake may be due to the presence of hospitals around the lake, as well as pollutants reaching this aquatic environment through the natural erosion of fields, water discharges and waterways.

Our matrices revealed a good source of Fe. In fact, the percentages of this element were of 92% for *M. galloprovincialis* and 169% for *T. decussatus*. After oral exposure, Fe induces a direct corrosive effect on the gastrointestinal tract. Additionally, it induces oxidative stress by altering mitochondrial oxidative phosphorylation, which may determine the severity of symptoms (Baranwal and Singhi, 2003). The primary effects are gastrointestinal, such as vomiting, diarrhoea, and bleeding. Other effects include dehydration and related symptoms such as hypotension, cardiovascular collapse, shock, hepatic necrosis and jaundice, coagulopathy, acute haemolytic anaemia, hemoglobinemia, haemoglobinuria, and death (Baranwal and Singhi, 2003). In addition, prolonged exposure can lead to an oxidative burst that may promote carcinogenesis in the gastrointestinal tract (Toyokuni et al., 2020). Concentrations of Ni were of 6% in *M. galloprovincialis* and 17% in *T. decussatus*. This percentage is optimal, based on the data obtained in the study conducted by Bartos et al. (2014), where the authors determined an ideal average nickel absorption rate of 16% related to the daily uptake. Good percentages were also obtained for Cu (42% for *M. galloprovincialis* and 17% for *T. decussatus*), Mn (24% for *M. galloprovincialis* and 53% for *T. decussatus*) and Zn (23% for *M. galloprovincialis* and 20% for *T. decussatus*).

## 4 Conclusion

Concentrations of essential and non-essential elements in the mussel *M. galloprovincialis* and the clam *T. decussatus* from the Faro Lake, located in Messina (Sicily), did not exceed the European Union and US Food and Drug Administration limits. Bivalves’ levels of elements can be determined by several matters, including their feeding, species, season, and environment. The results of this study highlight both the benefits and risks of consuming local mussels and clams and the importance of regularly testing locally produced bivalves for the presence of elements of toxicological concern and essential elements to protect consumer health. The data relating to the essential and toxic elements are within the maximum acceptable limits set by different health organizations and within the literature data. However, it would be required to set maximum amounts for all elements to facilitate the assessment of the safety or harmfulness of the bivalve samples. A daily consumption of 150 g was used to calculate the absorption rates of each element, and this demonstrated that mussels and clams are safe to consume. Using the samples as indicators of environmental pollution, our findings indicated that the samples have a high toxicological risk, for Cr in both *M. galloprovincialis* and *T. decussatus* and for Fe in *T. decussatus*. In fact, the Cr and Fe absorption in the samples analyzed is higher than 100%. However, to determine the risk of Cr, it would be appropriate to carry out further studies, especially to evaluate and control the levels of Cr (III) and Cr (VI) and to demonstrate the safety for humans. It remains to be understood today the Fe content in clams to understand its origin. As a result, this study also helped to assess the possible hazard to consumer health. The element concentrations observed in the two bivalve species from Lake Faro are generally lower than the tolerable weekly intake (TWI) values estimated as a risk to human health. Moreover, further studies will be conducted on seasonality in the concentrations of the elements determined in the bivalve samples could undergo changes and, consequently, the nutritional intake of the consumer.

## Data Availability

The original contributions presented in the study are included in the article/supplementary material, further inquiries can be directed to the corresponding authors.
